# 2.45 GHz Microwave Processing and Its Influence on Glass Fiber Reinforced Plastics

**DOI:** 10.3390/ma11050838

**Published:** 2018-05-18

**Authors:** Daniel Teufl, Swen Zaremba

**Affiliations:** Technical University of Munich, Chair of Carbon Composites, Boltzmannstr. 15, 85748 Garching, Germany; info@lcc.mw.tum.de

**Keywords:** microwave, 2.45 GHz, curing, composites, glass, epoxy, GFRP, mechanical properties, DIN EN 14130, hephaistos

## Abstract

During the production of fiber-reinforced composite materials, liquid resin is introduced into the fiber material and cured, i.e., hardened. An elevated temperature is needed for this curing. Microwave curing of composites has been investigated for some time, but it has mostly been done using small domestic or laboratory equipment. However, no investigation has been carried out using an industrial-sized chamber-microwave for glass fiber-reinforced plastic (GFRP). Here, we show that microwave curing produces laminates of the same quality as oven-cured ones. The study shows that, if the process is done right, GFRP samples can be produced with an industrial scale microwave. Even if not fully cured, microwave samples show a glass transition temperature measured with DMA (*T_g-DMA_*) that is comparable to the *T_g-DMA_* according to the proposed cure cycle on the data sheet. Specific microwave-cured configurations show better inter-laminar shear strength than oven specimens. The results show that microwave-based heat introduction can be a beneficial curing method for GFRP laminates. A microwave-optimized process is faster and leads to better mechanical properties.

## 1. Introduction

As Volker Mathes states in [1] “Fiber reinforced plastics—also known as composites—have been attracting enormous media interest over the last few months, especially in lightweight construction”. A sub-group of this fiber-reinforced plasticss (FRPs) are glass fiber reinforced thermosetting plastics (GRPs). GRPs make for the second biggest group of composite materials according to the market reports analyzed by Mathes; only short glass-fiber reinforced thermoplastics are used more. As a consequence, as Mathes continuous, the GRP production volumes and national economies can be related; thus, the total GRP market in Europe displays ‘only’ a slight growth of 1–2% over the last two years. So, on the one hand, GRP is undergoing a steady growth and is part of modern society. On the other hand, heating materials using microwave irradiation is common all over the world. In virtually every kitchen, microwaves are used to heat food faster than can be done using hotplates (conduction heating) or ovens (convection heating). A microwave’s electromagnetic waves penetrate the material to be heated and interact with it, heating its surface and core. This enables for homogeneous in-depth heating which, of course, is not restricted to food. The direct interaction and resulting volumetric heating offers potential for all industrial heat-transfer processes–for example, composite material heating. This is especially true for curing and post-curing of FRP, since they are often the most time- and energy-consuming process steps. Consequently, some research has been done in the field of 2.45 GHz microwave processing of epoxy, glass-composite, and carbon-composite materials since the 90 s. This research resulted in at least 43 publications including talks, letters, papers, project reports, and theses [2,3,4,5,6,7,8,9,10,11,12,13,14,15,16,17,18,19,20,21,22,23,24,25,26,27,28,29,30,31,32,33,34,35,36,37,38,39,40,41,42,43,44]. Divided into their main topics, these publications can be split into general [32,33,34], process [35,36,37,38,39,40,41,42,43,44], epoxy [15,16,17,18,19,20,21,22,23], carbon fiber-reinforced plastic (CFRP) [2,3,4,5,6,7,8,9,10,11,12,13,14], and GFRP [24,25,26,27,28,29,30,31] publications.

However, of the last three material categories and their 30 publications, most were done using small equipment like domestic microwaves [4,5,22,23,28], custom-build microwaves [6,7,16,19,20,21,24,25,27,29], laboratory microwaves [2,3,8,10,17,18,26], and not clearly defined but seemingly small equipment [9,15,31]. This small microwaves have all dimensions of about up to 30 × 30 × 20 cm${}^{3}$. Due to the fixed wavelength of 12.2 cm (2.45 GHz) in vacuum this small size is problematic; the field and consequently temperature distribution gets inhomogenous. The problem of a small applicator was discussed by Wallace in 2006 [23]. As a solution for the inhomogeneous field and temperature distribution, Wallace only manufactured small samples of 50 × 50 mm${}^{2}$. Another approach to handle the problem was chosen by Mooteri and Rao; they rotated their GFRP samples to reach a homogenous energy input [27,29]. While both approaches yield results on a small scale, they cannot—or only with limitations—scaled to industrial sized processes. For example, a rotating load prohibits connected lines.

**Figure 1:**
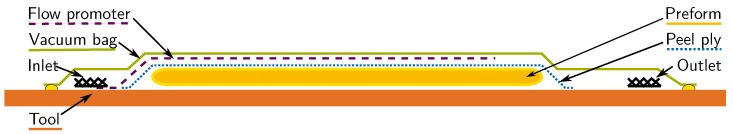
Infiltration setup used in this study. The vacuum applied to the outlet sucks in the resin that is kept at ambient pressure. The resin flows from the inlet through the flow promoter into the dry preform.

Consequently, of all publications only one study is known that characterizes GFRP and uses industrial size equipment. The study by Maenz et al. [30] uses a microwave chamber of 1400 × 1700 × 650 mm${}^{3}$ with 30 magnetrons in form of a press system. They manufacture GFRP samples with this microwave system and compare their mechanical properties to conventionally manufactured samples. No difference in mechanical properties between conventional and microwave processed samples is reported. Maenz et al. however, reheat their samples for 3 h at 100 ${}^{\circ }$C after microwave cure since they do not reach a homogeneous temperature distribution with their microwave process. Sumarizing, no publication is known to the authors that compares the mechanical properties of GFRP samples conventionaly manufactured with samples manufactured with microwave equipment suitable for industrial scale production. This paper, therefore, investigates the question whether an industrial-scale microwave applicator and vacuum-assisted half-mold process (Figure 1) does produce continuous glass fiber-reinforced plastic samples of the same quality as oven-processed ones. To answer this question, a *Vötsch Hephaistos 180/200* microwave applicator that is designed for industrial applications is used and slightly adapted. With this equipment and a corresponding industrial *Vötsch VTL 100/150* convection oven, sample plates are manufactured under comparable conditions. To do this, one resin system is cured with different cure cycles and identical set-up; thus, the difference between temperature and technology influences can be analyzed. The manufactured plates are investigated regarding their *T_g-DMA_* and inter-laminar shear strength tau (ILSS). The latter is done according to DIN EN ISO 14130 [45].

## 2. Materials, Equipment, and Methods

### 2.1. Materials

All data sheets and safety data sheets of the materials used will be made available at a request.

#### 2.1.1. Fiber Material

The fiber material used in this study is a standard E-Glass non-crimp-fabric (NCF) by Saertex (Saerbeck, NW, Germany) [46]. The +45/−45${}^{\circ }$ NCF (X-E-PB-627 g/m${}^{2}$-1270 mm) has a total areal weight of 627 g/m${}^{2}$. Its build-up consists of 300 g/m${}^{2}$ E-Glass in each 45${}^{\circ }$ and −45${}^{\circ }$ direction, 15 g/m${}^{2}$ epoxy binder on the topside, 3 g/m${}^{2}$ E-Glass in each 0${}^{\circ }$ respectively 90${}^{\circ }$ direction between the ±45${}^{\circ }$ layers, and 6 g/m${}^{2}$ polyethersulfone (PES) stitching (compare Appendix Table A3).

The 0${}^{\circ }$ NCF (U-E-PB-606g/m${}^{2}$-1200mm) has a total areal weight of 606 g/m${}^{2}$ with 520 g/m${}^{2}$ E-Glass in 0${}^{\circ }$ on top, 54 g/m${}^{2}$ E-Glass in 90${}^{\circ }$, 15 g/m${}^{2}$ epoxy binder on the bottom side, and 17 g/m${}^{2}$ pes stitching (compare Appendix Table A4).

The applied epoxy binder on both materials is Hexion Inc. (Columbus, OH, USA) [47] Epikote™Resin 05390. At time of the study, the binder was known as Momentive Specialty Chemicals Inc. (Columbus, OH, USA) Epikote Resin 05390.

#### 2.1.2. Resin System

Biresin^®^ CR 141, an anhydride epoxy resin from Sika (Stuttgart, BW, Germany)^®^ [48], was used as matrix material. The basic resin properties can be found in Table 1. This system was chosen because, according to experts from Sika, it has a lower toxicity and tolerates high heating rates much better than an amine system.

#### 2.1.3. Tooling Material

A requirement for volumetric heating in a microwave is a microwave-transparent or virtually transparent tooling material. To allow for comparison, glass plates and GFRP tools are used. Safety glass plates are used as tools for oven manufacturing. Glass-ceramic plates *NEXTREMA™724-8* by SCHOTT (Mainz, RP, Germany) [49] and GFRP tools are used as tools for microwave manufacturing.

### 2.2. Equipment

#### 2.2.1. Conventional Heating Equipment

Curing—and where applicable infiltration—of oven specimens takes place in a *Vötsch VTL 100/150* (Reiskirchen-Lindenstruth, HE, Germany) [50] convection oven which heats up to 250 ${}^{\circ }$C. A smaller convection oven, the *Haereus UT 12*(Reiskirchen-Lindenstruth, HE, Germany) [50] which heats up to 250 ${}^{\circ }$C is used to preheat the resin. To maintain resin temperature during degassing and infiltration, a *Haereus VT6130 M* vacuum oven is used.

#### 2.2.2. Microwave Equipment

**Figure 2:**
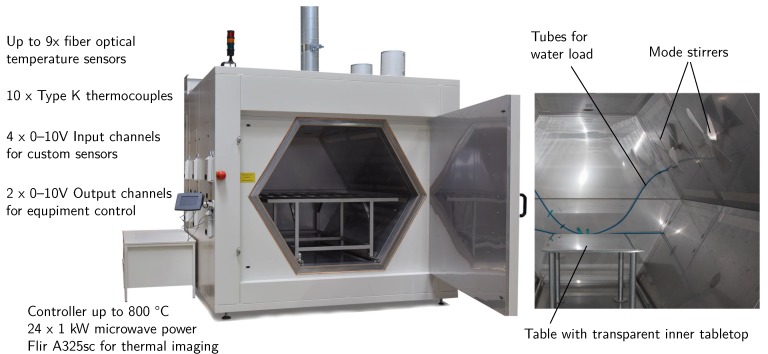
*Vötsch Hephaistos 180/200* located at the Chair of Carbon Composites with its capabilities and additions. The hexagon has a diameter of 1.8 m and a depth of 2 m.

The microwave utilized in this study is a *Vötsch Hephaistos 180/200* [51]) microwave applicator, see Figure 2. It has a inner hexagonal shape with a circumference of 1.8 m and a depth of 2 m. The hexagonal shape increases the field homogeneity according to [52,53]. A part introduced inside the microwave should not get to close to the walls of the hexagon to avoid arcing. With an arbitrary safety margin of 25 cm to each wall, flat parts of 1 × 1.5 m${}^{2}$ can be manufactured. This allows, for example, the manufacturing of different parts for aerospace [35] or any other applications that fit the size requirements. The applicator consists of two 1 m long modules, each of which is equipped with 12 magnetrons of nominal 1 kW each. These emit their power over a slit rectangular waveguide antenna into the process chamber. The magnetrons can be activated individually or in any combination. This provides a nominal power output of 1 to 24 kW. In addition, the power-output of the overall magnetron combination can be limited to a range of 10 to 100%. This is arranged by a pulsed operation of the magnetrons; therefore, they are only active for the given fraction of a 2 s interval; this is a hard-coded value in the microwave’s controller. The real maximum power output of a single magnetron is at approximately 0.85 kW. This maximum value has been measured using a calorimetric measurement set-up using a flowing water load provided bythe Karlsruher Institute of Technology (KIT) (Karlsruhe, BW, Germany) [54,55].

#### 2.2.3. Microwave Process Control

The above mentioned equipment’s correct absolute power output, however, has no practical relevance for the trials at hand; the power level is controlled by the part temperature over a proportional–integral–derivative (PID) control algorithm of the integrated controller and fiber-optic temperature sensors. The PID control parameters are important to reach a stable process. Experience from the KIT and Technical University of Munich (TUM) shows that a more homogeneous temperature distribution is reached if the power output variations, i.e., control oscillations, are minimized. For this reason, the PID parameters are adapted before and during plate production. For the same purpose, the amount of simultaneously active magnetrons is restricted, a constant water load is introduced, the magnetrons are constantly changed during the process, and mode stirrers are added to reach a stable process with homogeneous temperature distribution. A systematic study of these influencing factors is in progress.

#### 2.2.4. Temperature Measurement Equipment

The samples are supervised using an infrared *FLIR A325sc* camera (Wilsonville, OR, USA) [56] to check temperature homogeneity and to have visual feedback in case of thermal runaways during the microwave trials. Some samples were cured using added isolation. In this case, the infrared camera was used to detect irregularities in the process chamber. A fiber optic temperature measurement system by Optocon^®^ (Dresden, SN, Germany) [57] is used as temperature input for the PID controller. It consists of a *FOTEMPMK-19” Modular, fiber optic temperature monitoring system* with four 1-channel modules calibrated for renewable TS-NANO sensors. However, the TS-Nano sensor’s slow reaction time of up to 10 s makes adapting the PID parameters difficult. Nevertheless, the sensors are chosen due to their transparency to the microwave field and the possibility of refurbishing them. This makes it feasible for them to be placed under or in the laminate; after the process has been completed they are detached, tested and reused. If they have been damaged they are cut away and repaired.

### 2.3. Specimen Production

The following chapter describes the specimen production for the specimens with 300 × 480 mm${}^{2}$ and 3 mm thickness. A life datasheet (LDS) is maintained for every plate. This LDS is used to control process compliance against set standard procedures.

#### 2.3.1. Plate Manufacturing—Preforming

A binder-based preforming process was introduced, investigated, and optimized before specimen production. At the end of this optimization, a three step preforming process ensured constant conditions. First, layers are cut to 340 × 520 mm^2^, stacked with a lay-up of [(+/−45${}^{\circ }$), (0${}^{\circ }$/90${}^{\circ }$), (−/+45${}^{\circ }$), 90${}^{\circ }$, (+/−45${}^{\circ }$),(90${}^{\circ }$/0${}^{\circ }$), (−/+45${}^{\circ }$)], and sealed in a vacuum bag. The stacking can be seen in Figure 4 on page 8.

After stacking, the set-up is heated under full vacuum (<5 mBar) to 140 ${}^{\circ }$C for binder activation. This temperature is kept constant for 45 min to guarantee full heat-through before cooling. Last, the stabilized preform is stamped to 300 × 480 mm^2^ with rounded corners (r=40mm) using a stamping iron.

During the subsequent microwave processing, local electric conductive contaminations, such as carbon-fiber filaments, attract the microwave field and result in a much higher electrical field around the impurity; this leads to increased local heat development. Since the heat conduction in the laminate is comparably slow, the heat development results in local over-heating, which in turn damages the set-up. Therefore, a separate area in the chair’s workshop is used for the preform processing to avoid this type of contamination.

As mentioned, the described preforming process is a result of optimization. This took place after microsections of early manufacturing trials showed substantial voids. Early adaptions included variation in temperature, time and pressure during infiltration. However, only the manufacturing of a sample without binder showed no voids. Consequently, Louis Mahlau [58] investigated the preforming process and the binder material in a supervised term project. The PA1541A (Co-Polyamide web by *Spunfab* (Cuyahoga Falls, OH, USA) [59]), EPIKOTE™ Resin 05311 (Epoxy powder by *Momentive Specialty Chemicals Inc. (Columbus, Ohio, USA)* at the time of the study; by April 2018 distributed by *Hexion* [47]) binder materials and the original binder material ABE003 (Co-Polyester web by *AB-Tec* (Iserlohn, NW, Germany) [60]) where used. The preforming process was carried out using different compaction pressures (1, 15, and 100 bar) and heating technologies (oven, press). The preforming temperature was tested and fixed before Mahlau’s study. The compaction pressure did not appear to influence the porosity. The binder material was found to be the main factor influencing the porosity. As a consequence, glass-fiber material with 15 g/m${}^{2}$ predeposited epoxy binder EPIKOTE™ Resin 05390 (Hexion former *Momentive*) was used for the studies at hand. The EPIKOTE™Resin 05390 was chosen as a replacement for EPIKOTE™ Resin 05311, since the investigated binder 05311 was not available; Saertex recommended 05390 as a successor.

#### 2.3.2. Plate Manufacturing—Infiltration Process

Due to the resin’s high viscosity of 600 mPas at room temperature (RT), initial trials where carried out at an elevated infiltration temperature of 45 ${}^{\circ }$C. For microwave specimens, however, the set-up has to be transfered to the microwave equipment before the curing cycle starts. Since connecting the temperature sensors takes some time, the microwave specimens cool down between infiltration and curing. Consequently, the infiltration process was changed to RT for all configurations to maintain stable process conditions. The cycle with a maximum of 120 ${}^{\circ }$C (O_120) and the reference cycle (O_ref), however, had already been manufactured and tested. Thus, it was decided to repeat the the O_ref samples at RT to assess the infiltration temperature’s influence. Both processes are as follows:

**RT-Process:** The resin was mixed and degassed for 10 to 15 min. The inlet was opened until the plate is completely filled (≈10–20 min), after which the plate was flushed for 10 to 15 min. Subsequently, a pressure balance at 400 mBar (absolute) is set at the outlet and the inlet/resin-pot. This equalization pressure was held for 5 to 10 min. The vacuum connection was closed, the infiltrated plates were disconnected from the vacuum-equipment and transferred into the oven or microwave. Here, the temperature sensors were attached and connected to the microwave. Consequently, the cure cycle is started.

**45 ${}^{\circ }$C-Process:** The resin was mixed, heated to a temperature of 45 ${}^{\circ }$C in a convection oven to reduce the resin viscosity, degassed for 10 to 15 min in a vacuum oven, and kept at this temperature for infiltration. During this time, the tool and preform was pre-heated for one hour at 50 ${}^{\circ }$C and kept at this temperature during infiltration. The inlet was opened and after the plate was completely filled, which took about 5 to 10 min, the resin valve was kept open for another 5 min for flushing. Subsequently, a pressure balance at 400 mBar (absolute) was set at the outlet and the vacuum oven that contains the resin. This equalization pressure was held for 5 to 10 min before the infiltration was finished, the inlet and outlet were closed, and the cure cycle was started.

#### 2.3.3. Plate Manufacturing—Curing Processes

The curing process has a substantial influence on the properties of epoxy resins and thereby the investigated composite materials. On the one hand, the chemical reaction that is triggered during the curing process is an exothermic one. Thus, when the resin or composite is heated too fast over a certain point, the exothermic reaction results in a thermal runaway and the material overheats. To prevent this, a dwell time at 85 ${}^{\circ }$C is always maintained. On the other hand, if the maximum cure temperature is way below the resin system’s target $T_{g}$ of 139 ${}^{\circ }$C, the material does not cure completely. Consequently, the mechanical properties are not fully developed. To heed this possible influence of varying curing conditions on the mechanical properties, three different temperature profiles with maximum temperatures of 120 ${}^{\circ }$C, 140 ${}^{\circ }$C and 170 ${}^{\circ }$C are used for cure.

The same curing cycles will be used in oven and microwave curing; however, two slight adaptions are made in microwave processing. First, in microwave curing, the heat is generated directly in the material while there is a soaking time in oven curing. During oven curing it takes approximately 15 min until the part reaches the oven’s temperature. Consequently, the dwell time is reduced by this 15 min for microwave processes. Secondly, the microwave controller only takes the hottest temperature sensor into account. The average temperature in microwave processing, however, is slightly lower in all observed cases. As a result, the control temperature for microwave processes during the dwell time is increased by 2.5 ${}^{\circ }$C.

**Figure 3:**
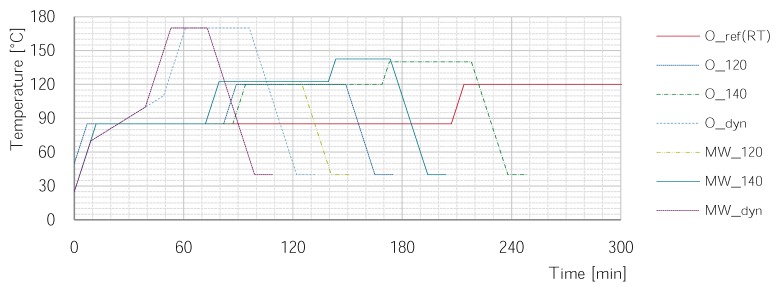
Different cure cycles used in this study. Microwave cycles have a dwell-time that is reduced by 15 min and a dwell temperature that is increased by 2.5 ${}^{\circ }$C.

With these adaptions, three different temperature cycles are compared; a fourth temperature cycle is used for reference in the oven only, see Table 2 and Figure 3.

The O_ref is the cure cycle used to obtain the mechanical and thermal properties of the resin stated in the datasheet. It is 3 h at 80 ${}^{\circ }$C, 120 ${}^{\circ }$C, and 140 ${}^{\circ }$C. Due to its length of over 10 h it is only investigated in the oven The first comparing temperature cycle (O_120 / MW_120) ends after a shortened 120 ${}^{\circ }$C dwell time, resulting in an incompletely cured resin. This is done to enhance possible effects of microwave curing that occur before full cure. The second cure cycle (O_140/M_140) has a longer 120 ${}^{\circ }$C phase and adds a short 140 ${}^{\circ }$C cure phase. Through the 140 ${}^{\circ }$C addition, the degree of cure shall be pushed nearly to the limit. The last comparing cycle (O_dyn/MW_dyn) is carried out with the intention of microwave optimization; a steady temperature rise up to 170 ${}^{\circ }$C is used to potentially utilize the in-depth heating of microwaves. To take the exothermic reaction into account, a very slow heating rate of 1${}^{\circ }$C/min starting at 70 ${}^{\circ }$C is applied. This slow heating rate is maintained up to 100 ${}^{\circ }$C in the microwave and, taking the soaking time into account, up to 110 ${}^{\circ }$C in the oven.

### 2.4. Quality Control and Testing

#### 2.4.1. Quality Control

**Figure 4:**
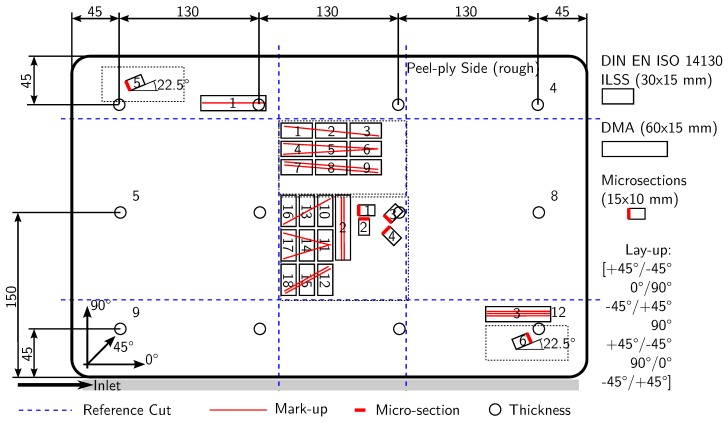
Positions of thickness measurements, samples for quality control, and ILSS samples (Measurements in mm).

After manufacturing, the thickness of every plate is measured at 12 distinct points to control the thickness before specimen preparation. During specimen production, up to 6 micro-sections are prepared under different angles and at varying positions to check for voids. See Figure 4 for measurement points and specimen positions.

#### 2.4.2. $T_{g-DMA}$ Measurements

Three DMA measurements are carried out for every manufactured and tested plate to obtain the $T_{g-dma}$ and to obtain an indication of the degree of cure. The double cantilever tests performed have a clamping length of 50 mm and use specimens that are 60 × 15 mm${}^{2}$ long and 3 mm thick. The specimens are heated from 70 ${}^{\circ }$C to 170 ${}^{\circ }$C with 2 ${}^{\circ }$C/min while a constant load of ±10 µm is applied at 1 Hz. The peak of the tan(δ) curve is used as *T_g-DMA_*. For the ease of reading, *T_g_* will be used for *T_g-DMA_* in this paper. The set specimen positions are shown in Figure 4. The equipment used is a *Q800 DMA* of *Texas Instruments*.

#### 2.4.3. Interlaminar Shear Strength Investigation

The inter-laminar shear strength (ILSS) tests are based on DIN EN 14130 [45]. For the tests, a universal testing machine Hegewald and Peschke electromechanical drive with a 10 kN class 1 load cell is used. The test speed is set to 1 mm/min. The radii of the test rick follow the standard; the supports have 2 mm radii, the pressure fin has a radius of 5 mm. A supporting width of 17 mm (5 times the nominal sample thickness plus 2 mm) is used for all tests; the samples break due to shear. Before the test a caliper is used to measure the width and thickness out to two digits of each specimen at three points; the mean values are used for calculating the apparent shear strength. Samples where one of the measured thickness values deviates more than ±0.2 mm of the series mean thickness are tested but excluded afterward. A maximum of 9 and a minimum of 6 samples of 30 mm to 15 mm are tested for every plate in each 0${}^{\circ }$ (A) and 90${}^{\circ }$ (B) direction. Each sample is placed on the test rig with peel-ply side pointing in pressure fin direction. Recording starts when an initial load of 5 N is reached. For the evaluation, the apparent shear strength *τ_ILSS_* is calculated. All samples that underwent a specific cure temperature-profile are averaged and the according 95% confidence interval is calculated.

## 3. Results and Discussion

In the four sections of results and discussion, the first section reflects on some lessons learned during preforming and microwave processing. The second section assesses the influence of the infiltration temperature. The third and fourth section compare the glass transition temperature (*T_g_*) development and ILSS properties for various cycles and technologies.

### 3.1. Processing of Microwave Specimens

#### 3.1.1. Preforming of Microwave Specimens

A carbon-fiber-free environment is necessary to manufacture GFRP samples on a transparent tool with no additional absorbers; the environment used in this study was not enough to achieve this. After process optimizations, samples up to 120 ${}^{\circ }$C were manufactured without problems. However, at higher temperatures more power is needed; burnings occurred that were traced back by their appearance to entrapped carbon-fibers.

Internal investigations showed that the heat introduced into a filament strongly depends on filament length and the microwave power level. Consequently, at a high power level that depends on the filament length, the impurity heats up and locally burns the matrix. The burned matrix is a good absorber and heats up further. A factor additionally influencing this carbon-fiber burnout is the ongoing cure of the GFRP. On the one hand, more energy input is needed to reach higher temperatures due to heat-transfer effects. On the other hand, the dielectric properties of the matrix change and the energy conversion inside the material is reduced. Thus, to maintain a constant heating rate or temperature, the microwave power is increased by the controller. The higher the microwave power, the more energy is transferred to a filament; shorter filaments that are entrapped get critical, overheat, and burn the set-up. This is especially problematic for the set-up at hand where nearly no energy is transferred directly to the tool. If the tool itself did absorb some energy and therefore heat up under irradiation, the energy input needed in the GFRP would be reduced.

#### 3.1.2. Curing Process in the Microwave

**Figure 5:**
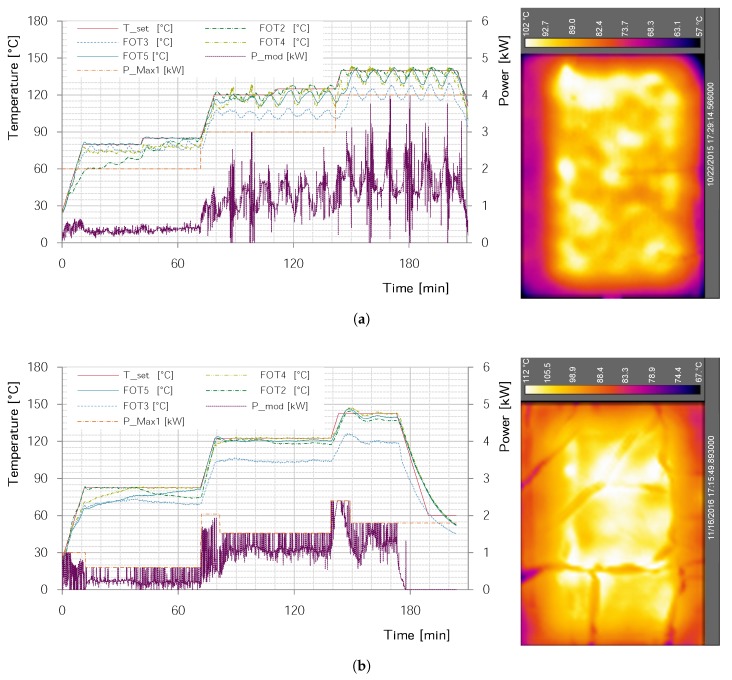
Microwave temperature and power log with thermal images at approximately 165 min. (**a**) Early stage of process with water load but without mode stirrers or adapted control parameters. Strong control oscillations occur and temperature homogeneity is poor; (**b**) Adapted process with water load, mode stirrers and adapted control parameters. Control oscillations are minimizend and temperature homogeneity is increased.

Adapting control parameters was most beneficial for reaching a stable process and good specimens, as can be seen in Figure 5. Since the exothermic reaction, the change of dielectric properties during the cure, and the influence of the set-up (tooling material, plate thickness etc.) is highly specific, it would be impossible to get universal rules out of the one investigated set-up. Thus, no specific investigation was carried out in this regard, but rather, the process was adapted step-by-step. The following, therefore, can only give a slight insight.

In comparison to the first trials, only the integral and differential factors have been increased and adapted. It is believed that this mainly had to be done due to the high response time of the used temperature sensors. While the energy input and, therefore, temperature gradient changes instantly with the power level, the temperature sensors lack behind in their true value. A higher differential factor reacts stronger to the gradient of the temperature measurements—which occurs earlier if not instantly—and counteracts the delayed value change and overshoot. The higher integral value likewise helps to make the system react slower. However, while the higher integral value makes the system more stable at temperature steps below 120 ${}^{\circ }$C, it results in a behavior that is too slow at higher temperatures. Heat radiation and transfer results in faster cooling or slower heating, see Figure 5 bottom 120–140 ${}^{\circ }$C. As a possible solution for this problem, Promaglaf^®^-HTI 1100 insulation plates by Promat (Ratingen, NW, Germany) [61] were used surrounding the set-up in form of a box. While this decreased the necessary microwave power and enhanced temperature homogeneity, it also obscured the set-up from thermal imaging. The practice was canceled after a smoldering fire—probably due to a carbon fiber contamination—was detected only after serious smoke development.

Apart from the equipment’s integrated PID control parameters, at least four factors are essential for stable microwave processing the GFRP-plates. First and foremost, the number of magnetrons is limited according to the needed heating power. This, however, contradicts a need to use as many sources as possible to generate a chaotic field. Second, to be able to use more than one magnetron per module, a constant water flow through an 8 mm tube in the back of the microwave is maintained; the %-power level to reach the target temperature is shifted to higher values. Without this, the PID controller reaches a lower limit of 10% since the curing of the GFRP plate only needs a few hundred Watt. Third, to homogenize the field through more “chaos”, the active 2, 3, or 4 magnetrons per module are changed every 10 s. Finally, slowly turning reflective fans—i.e., mode stirrers are added to reach an assumable, constantly changing electromagnetic field. As a result of these four changes and the adaption of the control parameters, the power input and temperature distribution over the plate gets more homogeneous and constant, compare Figure 5.

It can be assumed that the tooling material has no influence on the process or the test results. It was observed that there is a difference in the heat-up characteristic of GFRP tools and glass-ceramic tools; the GFRP heats up slightly while the glass-ceramic stays cold. Consequently, the temperature gradient between the manufactured plate’s edge and the tool is lower for the GFRP tool. However, this only changes the temperature distribution approximately 3 cm around the edge. An influence on the test specimen’s properties by this can be ruled out due to their position, compare Figure 4. Furthermore, no indication for an influence of the tooling material on the test results could be found during the evaluation.

### 3.2. Influence of Start Temperature—Comparison of Reference Plates

While the infiltration at 45 ${}^{\circ }$C takes less than 5 min, 15 to 20 min are needed to infiltrate at RT. The high viscosity of the resin leads to the high infiltration times; since the pot-life of the resin is above 24 h at RT, this is not a problem.

To assess the influence of start and infiltration temperature on the mechanical properties, independent-sample, double sided *t*-tests are conducted (α=0.05). The *t*-test’s results are stated in the form (t(degreesoffreedom)=t-value,p=significancelevel,d=Cohen’sd). First, RT and 45 ${}^{\circ }$C infiltrated samples cured with the reference cycle are compared. A significant difference can be seen for *T_g_* (t(5)=5.37,p=0.003,d=2.99) and apparent shear strength in 90${}^{\circ }$-direction (t(31)=2.16,p=0.039,d=0.22). While the difference in *T_g-DMA_* between RT and 45 ${}^{\circ }$C infiltrated reference plates is significant and Cohen’s d is high, the actual difference is only 0.77 ${}^{\circ }$C. The mismatch is below common variations in *T_g-DMA_* measurements and could be due to some constant variation on the different DMA test dates. The difference of 1.29 MPa between the $\tau_{ILSS-B}$ is way smaller than each standard deviation (SD) (SD${}_{Ref-RT}=2.1\;\mathrm{MPa}$, SD${}_{Ref-45}=1.73\;\mathrm{MPa}$). This can likewise be seen in the small effect strength of d=0.22. A summary of the mean values and statistics can be found in Table 3. Due to the small difference, both reference cycles will be seen as one for the following comparison. Two RT samples apparent interlaminar shear strength either in 0° (A) or 90° (B) direction (*τ_ILSS_*) deviated more than two standard deviations downward from their plates mean value (RG-t3-22-IS-A-05 ($\tau_{ILSS-A}=36.4\;\mathrm{MPa}$); RG-t3-20-IS-B-11 ($\tau_{ILSS-B}=42.0\;\mathrm{MPa}$). This specimens were classified as outliers and excluded from the above and following evaluation.

### 3.3. Development of $T_{g}$ for Different Cure Cycles and Heating Methods

**Figure 6:**
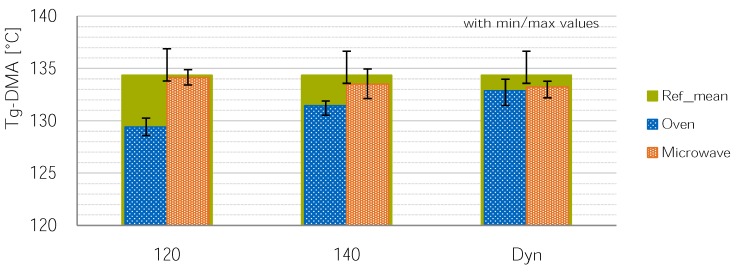
*T_g_* comparison of different cure cycles and methods. Since only three measurements for MW_dyn exist, the Min-Max values are given as error bars instead of SD.

The reference cure cycles according to the resin’s datasheet yields a *T_g_* of 134.6 ${}^{\circ }$C and 133.8 ${}^{\circ }$C. As expected, the *T_g_* of the O_120 cycle is the lowest at 129.4 ${}^{\circ }$C (SD=0.57), 4.4 ${}^{\circ }$C below reference. The $T_{g}$ of the O_140 is slightly higher at 131.44 ${}^{\circ }$C(SD=0.52) followed by the dynamic 170 ${}^{\circ }$C cycle with a *T_g_* of 133.87 ${}^{\circ }$C (SD=0.75). The MW_120 cure cycle, however, yields a *T_g_* of 134.1 ${}^{\circ }$C (SD=0.43) that lies between the two reference configurations. The *T_g_* of MW_140 and MW_dyn lie slightly below with 133.5 ${}^{\circ }$C (SD=1.22) and 132.2 ${}^{\circ }$C (SD=n.g.). All values are listed in Table 4 and Figure 6.

One possible explanation for the resin’s behavior at 120 ${}^{\circ }$C microwave cure can be found in studies investigating the influence of microwave curing on the reaction path of epoxy resins. This phenomenon was investigated in greater depth by Marand in 1992 [62], by Wei in 1995 [63], and by Wallace in 2005 [23]. These studies show that microwave curing may change the rate of cross-linking due to the change in reaction path. For example, “the epoxy-amine reaction is more dominant in the microwave-cured samples than the other possible curing reactions including the epoxy-hydroxyl reaction” while curing PR500 by 3 M [23]. It can be assumed that similar effects occur during the curing of the present anhydride system. One study conducted by Tanrattanakul in 2005 [22] for a anhydride system is ignored since the present set-up is temperature controlled. In the mentioned investigation, “the applied [microwave] power was based on the physical performance of the cured samples. No air bubbles and no burning were criteria for good specimens”. The criteria bubbles and burning would only be met at very high temperatures in the resin. Thus, it can be assumed that the microwave curing in Tanrattanakul’s study still took place at much higher temperatures than the oven curing. This would most probably result in higher *T_g_* values independent of other influencing factors. Apart from this, the authors know off no in-depth studies that compare the reaction paths of anhydride systems undergoing microwave and conventional cure.

**Figure 7:**
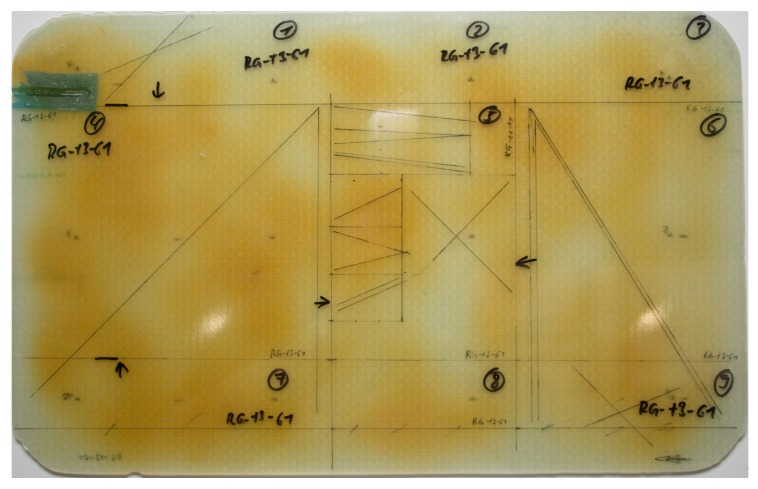
Sample plate manufactured in the microwave with bad temperature homogeneity during cure (compare Figure 5 top) results in different colored areas.

In summary, the *T_g_* of the oven specimens develops as expected while the microwave specimens show the reference cycles *T_g_* after only 30 min cure at 120 ${}^{\circ }$C. This can also be confirmed by a double sided, independent-sample *t*-test between MW_120 and O_ref-mean (t(18)=0.82p=0.42). In contrast to the tg, the visual appearance differs between O_ref and MW_120 cured samples. The resin system that was used turns a brown to reddish tone for all cure cycles exceeding 140 ${}^{\circ }$C; the MW_120 and other 120 ${}^{\circ }$C-cured plates have a cloudy-white appearance. This change in color is also visible in early microwave-cured samples with a high temperature variance, compare Figure 7.

### 3.4. ILSS Properties

In addition to a simple comparison of the results, double sided, independent-sample *t*-tests are conducted (α=0.05) between the results of cycles to determine whether the difference is statistically significant before interpretation.

#### 3.4.1. Number of Specimens and Modes of Failure

**Figure 8:**
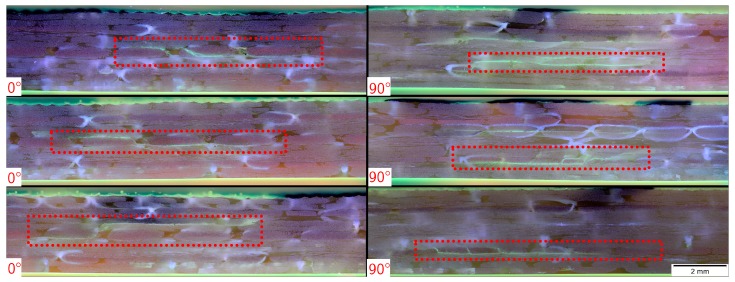
Microsection of failed ILSS specimens in both directions with marked-up crack areas.

For each process configuration, at least 2 plates, each with up to 9 specimens in 0${}^{\circ }$ and 90${}^{\circ }$-direction were tested. The minimum number of samples was tested for the 140 ${}^{\circ }$C-oven cycle for which only two plates could be used. ILSS Values in the 90${}^{\circ }$direction are about 10% higher than in 0${}^{\circ }$- direction. The 90${}^{\circ }$ samples with parallel orientation to the innermost symmetry layer (4) fail away from the inner area in the area of layer (3) or (5). The 0${}^{\circ }$ samples show a failure mode where the crack is more central around layer (4); the initial point of failure is most likely to occur in the center area where the shear stress is highest, see Figure 8. Independent from this difference, all samples show shear-induced failure.

#### 3.4.2. Oven Specimen Behavior

**Figure 9:**
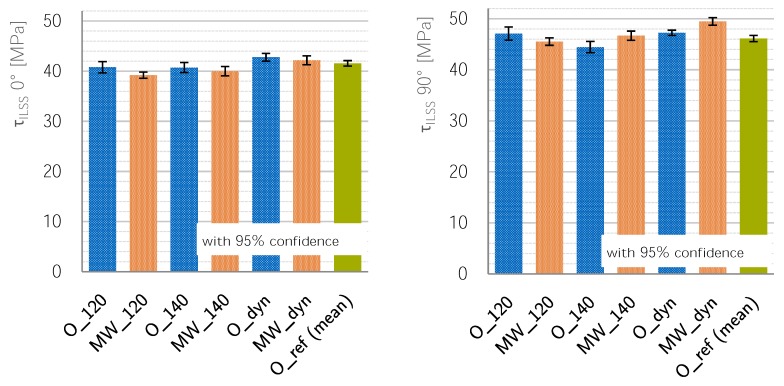
$\tau_{ILSS}$ in 0${}^{\circ }$ to the left and 90${}^{\circ }$ to the right with 95% confidence interval.

All samples behave in the same manner when comparing the temperature cycles for both orientations in each heating system. The order changes when comparing oven and microwave specimens. For oven specimens, the O_120 samples show a similar *τ_ILSS_* compared to the reference samples. The strength of O_140 samples in both directions is lower, but only significant in the 90${}^{\circ }$-direction with a delta of ≈1.7 MPa to the reference cycle; the O_dyn results are both ≈1.2 MPa higher compared to the reference cycles. In summary, the 140 ${}^{\circ }$C-cycle in B direction shows a drop in apparent shear strength while the dynamic oven cycle beats the properties of the reference cycle in both directions, see Figure 9.

#### 3.4.3. Microwave Specimen Behavior

For microwave specimens, the 120 ${}^{\circ }$C samples have the lowest strength, followed by the MW_140 and the MW_dyn samples, see Figure 9. Apart from the difference between MW_120 and MW_140 in 0${}^{\circ }$direction, all differences are significant, compare Table A2 in the Appendix A.

#### 3.4.4. Comparison between Oven and Microwave Specimens

For the 120 ${}^{\circ }$C samples, the microwave samples in both directions have a lower shear strength; both orientations show a significant difference with a delta of 1.6 MPa between their means. For both other temperature cycles, the 0${}^{\circ }$ tests show no significant difference between oven and microwave samples. The 90${}^{\circ }$ tests, however, show a clear and significant difference where the MW samples have an apparent shear strength $\tau_{ILSS-B}$ that is ≈2.2 MPa higher than the oven specimens’, see Table 5. Appendix A of Table A1 shows a table comparing oven and microwave cycles and their comparing *t*-tests.

## 4. Conclusions

Samples were manufactured using four different cure cycles by means of oven and microwave heating that ranged from 120 ${}^{\circ }$C to 170 ${}^{\circ }$C. Industrial sized equipment was used throughout the study. While some adaptions were necessary for microwave processing, the investigation showed that the ILSS properties of the microwave-cured samples are comparable or better than oven-cured samples.

While an industrial microwave was used for specimen manufacturing, some adaptions were essential to get a stable process. The small load made an added dead-load necessary; mode-stirrers increased temperature homogeneity and are believed to be a useful addition in any circumstance. The process control for microwave curing, furthermore, is very complex compared to simple oven curing; this is due to the direct microwave-matter interaction. A more sophisticated control software would be welcome for research purposes; for industrial use, this is not considered necessary after a process is defined. The adaptions introduced provide a benefit for further developments.

The *T_g-DMA_* of oven-cured samples develops as expected starting at 129.4 ${}^{\circ }$C for the 120 ${}^{\circ }$C cure cycle up to 134.6 ${}^{\circ }$C for the reference cycle. However, the microwave-cured samples’ *T_g-DMA_* is consistently just as high as the reference cycles. This is believed to be due to another reaction path during microwave curing as is supported by other studies. The apparent shear strength of 120 ${}^{\circ }$C cured microwave specimens is lower than that of oven-cured samples; all other configurations, however, yield similar or better results for microwave curing. The results of configurations cured using a dynamic-cure cycle—with only ramps and lacking any dwell-time—consistently beat the reference cycle. The dynamic microwave-cured samples beat the reference cycle in 90${}^{\circ }$-orientation by 3.4 MPa or 7%. It can be said that microwave processing can improve the mechanical properties.

It was shown that microwave curing within a large scale applicator produces glass-fiber-reinforced plastic laminates with a quality similar to oven-cured samples. Furthermore, the optimization of the process for microwave curing reduces the cycle time and yields samples with better ILSS properties.

However, the *T_g-DMA_* development deviates from the oven process; the microwave samples develop a *T_g-DMA_* comparable to the 140 ${}^{\circ }$C reference-cycle of ≈134 ${}^{\circ }$C at 120 ${}^{\circ }$C cure temperature. This shows that there is some difference in microwave and oven curing.

## Figures and Tables

**Table 1:** Properties of Biresin CR141 according to its datasheet.

Mixture			Neat Resin Specimen *		
Property	Value	Unit	Property	Value	Unit
Potlife, 100 g/RT	>24	h	Tensile strength	78	MPa
Mixed viscosity, 25 ${}^{\circ }$C	600	mPa.s	Tensile E-Modulus	3200	MPa
Mixed viscosity, 50 ${}^{\circ }$C	100	mPa.s	Elongation at break	3.3	%
(50 ${}^{\circ }$C acc. to viscosity curve provided by Sika)			Impact resistance	18	kJ/m^2^
			*T_g_* (ISO 11357)	139	${}^{\circ }$C

* approx. Values after 3 h/80 ${}^{\circ }$C + 3 h/120 ${}^{\circ }$C + 3 h/140 ${}^{\circ }$C.

**Table 2:** Different cure cycles with starting and infiltration temperatures used in this study. 5 ${}^{\circ }$C/min heating and cooling ramps are used if not given otherwise.

Start	RT	50 ${}^{\circ }$C	85 ${}^{\circ }$C (min)	120 ${}^{\circ }$C (min)	140 ${}^{\circ }$C (min)	170 ${}^{\circ }$C (min)	Cycletime (min)
**O_120**	No	Yes	75	60	-	-	170
**MW_120**	Yes	No	60	45	-	-	141
**O_140**	Yes	No	75	75	45	-	238
**MW_140**	Yes	No	60	60	30	-	194
**O_dyn**	Yes	No	70 ${}^{\circ }$C to 110 ${}^{\circ }$C with 1 ${}^{\circ }$C/min			35	122
**MW_dyn**	Yes	No	70 ${}^{\circ }$C to 100 ${}^{\circ }$C with 1 ${}^{\circ }$C/min			20	99
**O_ref**	Yes	Yes	195	195	195	-	628

**Table 3:** Values and test statistic of independent-sample *t*-tests for different infiltration temperatures.

Property		M${}_{O_{\mathrm{Ref}-\mathrm{RT}}}$	SD${}_{O_{\mathrm{Ref}-\mathrm{RT}}}$	M${}_{O_{\mathrm{Ref}-45}}$	SD${}_{O_{\mathrm{Ref}-45}}$	*t*-Test
tg	(${}^{\circ }$C)	134.59	0.192	133.82	0.271	t(5)=5.37,p=0.003,d=2.99
$\tau_{ILSS-A}$	(MPa)	42.15	1.53	41.38	1.44	t(31)=1.60,p=0.120,d=1.07
$\tau_{ILSS-B}$	(MPa)	46.88	2.1	45.59	1.67	t(31)=2.16,p=0.039,d=0.22

**Table 4:** Average $T_{g-dma}$ values and their statistics.

		O_120	O_140	O_dyn	O_ref	MW_120	MW_140	MW_dyn
**Mean**	(${}^{\circ }$C)	129.4	131.4	132.9	134.4	134.1	133.5	133.2
**min**	(${}^{\circ }$C)	128.6	130.5	131.5	133.6	133.4	132.1	132.2
**max**	(${}^{\circ }$C)	130.3	131.9	134	136.7	134.9	135	133.8
**Count**	(-)	8	6	9	14	12	5	3
**SD**	(${}^{\circ }$C)	0.57	0.52	0.75	0.98	0.43	1.22	n.g.
**95%-conf**	(${}^{\circ }$C)	0.47	0.55	0.58	0.56	0.27	1.52	2.23

**Table 5:** Test results of ILSS trials.

		O_120	MW_120	O_140	MW_140	O_dyn	MW_dyn	O_ref
$\tau_{ILSS-A}$	(MPa)	40.8	39.2	40.7	40.0	42.8	42.2	41.7
95% Conf.	(MPa)	1.13	0.63	1.00	0.94	0.78	0.90	0.48
$\tau_{ILSS-B}$	(MPa)	47.1	45.5	44.4	46.7	47.3	49.5	46.1
95% Conf.	(MPa)	1.29	0.73	1.11	0.90	0.51	0.73	0.60

## Appendix A

**Table A1:** Test results and statistics of ILSS Trials in (A) 0${}^{\circ }$ direction and (B) 90${}^{\circ }$ direction; oven-microwave comparison.

**Orientation** **0°**	$\tau_{\mathrm{ILSS}-A}$ **(MPa)**	**Count** **(−)**	**SD** **(MPa)**	**95% Conf.** **(MPa)**	***t*-Test** **Row to Row Above**
O_120	40.8	14	1.95	1.13	
MW_120	39.2	26	1.56	0.63	t(22)=2.6,p=0.016,d=0.92
O_140	40.7	18	2.00	1.00	
MW_140	40.0	27	2.37	0.94	t(40)=1.1,p=0.279
O_dyn	42.8	20	1.68	0.78	
MW_dyn	42.2	19	1.86	0.90	t(36)=1.05,p=0.3
O_ref (mean)	41.6	41	1.70	0.54	
**Orientation** **90°**	$\tau_{\mathrm{ILSS}-B}$ **(MPa)**	**Count** **(−)**	**SD** **(MPa)**	**95% Conf.** **(MPa)**	***t*-Test** **Row to Row Above**
O_120	47.1	17.0	2.51	1.29	
MW_120	45.5	20.0	1.57	0.73	t(26)=2.22,p=0.035,d=0.76
O_140	44.4	16.0	2.08	1.11	
MW_140	46.7	25.0	2.18	0.90	t(33)=3.32,p=0.002,d=1.05
O_dyn	47.3	26.0	1.27	0.51	
MW_dyn	49.5	18.0	1.48	0.73	t(33)=5.2,p<0.001,d=1.64
O_ref (mean)	46.1	43.0	1.95	0.60	

The *t*-test’s results are stated in the form (t(degreesoffreedom)=t-value,p=significancelevel,d=Cohen’sd).

**Table A2:** Test results and statistics of ILSS Trials in (A) 0${}^{\circ }$ direction and (B) 90${}^{\circ }$ direction sorted according to cure temperature and method with test statistics.

**Orientation** **0°**	$\tau_{\mathrm{ILSS}-A}$ **(MPa)**	**Count** **(−)**	**SD** **(MPa)**	**95% Conf.** **(MPa)**	***t*-Test** **Row to Row Above**
O_120	40.8	14	1.95	1.13	
O_140	40.7	18	2.00	1.00	t(28)=0.08,p=0.937
O_dyn	42.8	20	1.68	0.78	t(33)=3.40,p=0.002,d=1.12
O_ref (mean)	41.6	41	1.70	0.54	t(35)=2.44,p=0.020,d=0.69
MW_120	39.2	26	1.56	0.63	
MW_140	40.0	27	2.37	0.94	t(45)=1.45,p=0.153
MW_dyn	42.2	19	1.86	0.90	t(43)=3.49,p=0.001,d=1.00
O_ref (mean)	41.6	41	1.70	0.54	t(30)=0.99,p=0.329
**Orientation** **90°**	$\tau_{\mathrm{ILSS}-B}$ **(MPa)**	**Count** **(−)**	**SD** **(MPa)**	**95% Conf.** **(MPa)**	***t*-Test** **Row to Row Above**
O_120	47.1	17	2.51	1.29	
O_140	44.4	16	2.08	1.11	t(31)=3.32,p=0.002,d=1.15
O_dyn	47.3	26	1.27	0.51	t(22)=4.88,p<0.001,d=1.74
O_ref (mean)	46.1	43	1.95	0.60	t(67)=2.88,p=0.005,d=0.65
MW_120	45.5	20	1.57	0.73	
MW_140	46.7	25	2.18	0.90	t(43)=2.05,p=0.046,d=0.59
MW_dyn	49.5	18	1.48	0.73	t(41)=5.01,p<0.001,d=1.45
O_ref (mean)	46.1	43	1.95	0.60	t(42)=7.30,p<0.001,d=1.83

The *t*-test’s results are stated in the form (t(degreesoffreedom)=t-value,p=significancelevel,d=Cohen’sd).

**Table A3:** Architecture of ±45${}^{\circ }$ NCF X-E-PB-627g/m^2^-1270 mm according to datasheet by Saertex.

	Build-Up	Areal weight (g/m^2^)	Tolerance (±%)	Material
**Top**	**Powder**	15	20	Momentive Epikote Resin 05390
	**45${}^{\circ }$**	300	5	E-Glass 300 tex
	**90${}^{\circ }$**	3	5	E-Glass 68 tex
	**0${}^{\circ }$**	3	5	E-Glass 68 tex
**Bottom**	**−45${}^{\circ }$**	300	5	E-Glass 300 tex
	**Stitching**	6	±1 g/m${}^{2}$	pes 76 dtex

**Table A4:** Architecture of 0${}^{\circ }$ NCF U-E-PB-606g/m^2^-1200 mm according to datasheet by Saertex.

	Build-Up	Areal weight (g/m^2^)	Tolerance (±%)	Material
**Top**	**0${}^{\circ }$**	520	5	E-Glass 1,200 tex
	**90${}^{\circ }$**	54	5	E-Glass 68 tex
**Bottom**	**Powder**	15	20	Momentive Epikote Resin 05390
	**Stitching**	17	±3 g/m${}^{2}$	pes 76 dtex
